# Development and Validation of a New Questionnaire Assessing Quality of Life in Adults with Hypopituitarism: Adult Hypopituitarism Questionnaire (AHQ)

**DOI:** 10.1371/journal.pone.0044304

**Published:** 2012-09-11

**Authors:** Hitoshi Ishii, Akira Shimatsu, Yasuhiko Okimura, Toshiaki Tanaka, Naomi Hizuka, Hidesuke Kaji, Kunihiko Hanew, Yutaka Oki, Sayuri Yamashiro, Koji Takano, Kazuo Chihara

**Affiliations:** 1 Department of Endocrinology, Tenri Hospital, Tenri, Japan; 2 Clinical Research Institute, National Hospital Organization, Kyoto Medical Center, Kyoto, Japan; 3 Department of Nutrition, Metabolism and Food Science, Graduate School of Life Science, Kobe Women’s University, Kobe, Japan; 4 Tanaka Growth Clinic, Tokyo, Japan; 5 Department of Medicine II, Tokyo Women’s Medical University, Tokyo, Japan; 6 Division of Physiology/Metabolism, University of Hyogo, Akashi, Japan; 7 Hanew Endocrine Clinic, Sendai, Japan; 8 Department of Medicine, Hamamatsu University School of Medicine, Hamamatsu, Japan; 9 Department of Nephrology and Endocrinology, the University of Tokyo Faculty of Medicine, Tokyo, Japan; 10 Department of Diabetes and Endocrinology, Hyogo Prefectural Kakogawa Medical Center, Kakogawa, Japan; University of California at San Diego, United States of America

## Abstract

**Objective:**

To develop and validate the Adult Hypopituitarism Questionnaire (AHQ) as a disease-specific, self-administered questionnaire for evaluation of quality of life (QOL) in adult patients with hypopituitarism.

**Methods:**

We developed and validated this new questionnaire, using a standardized procedure which included item development, pilot-testing and psychometric validation. Of the patients who participated in psychometric validation, those whose clinical conditions were judged to be stable were asked to answer the survey questionnaire twice, in order to assess test-retest reliability.

**Results:**

Content validity of the initial questionnaire was evaluated via two pilot tests. After these tests, we made minor revisions and finalized the initial version of the questionnaire. The questionnaire was constructed with two domains, one psycho-social and the other physical. For psychometric assessment, analyses were performed on the responses of 192 adult patients with various types of hypopituitarism. The intraclass correlations of the respective domains were 0.91 and 0.95, and the Cronbach’s alpha coefficients were 0.96 and 0.95, indicating adequate test-retest reliability and internal consistency for each domain. For known-group validity, patients with hypopituitarism due to hypothalamic disorder showed significantly lower scores in 11 out of 13 sub-domains compared to those who had hypopituitarism due to pituitary disorder. Regarding construct validity, the domain structure was found to be almost the same as that initially hypothesized. Exploratory factor analysis (n = 228) demonstrated that each domain consisted of six and seven sub-domains.

**Conclusion:**

The AHQ showed good reliability and validity for evaluating QOL in adult patients with hypopituitarism.

## Introduction

Hypopituitarism is generally a chronic and life-long disease involving deficiencies in one or more of the six pituitary hormones: growth hormone (GH), thyroid stimulating hormone (TSH), adrenocorticotrophic hormone (ACTH), luteinizing hormone (LH), follicle stimulating hormone (FSH) and prolactin (PRL). It is also occasionally associated with diabetes insipidus caused by deficiency of antidiuretic hormone (ADH) [1]. Hypopituitarism and diabetes insipidus can result from various conditions affecting the pituitary and/or the hypothalamus, including tumors, infiltrative lesions, infarction, apoplexy, trauma, and infection. It can also be caused by breech delivery with asphyxia, surgery, and radiation therapy. Hypopituitarism and diabetes insipidus in adults may result in various symptoms, such as fatigue, loss of energy, decreased muscle strength, decreased sociability, emotional instability, sexual dysfunction, hypotension, polyuria, polydipsia, and disturbance of consciousness and cognitive function. These symptoms cause deterioration in the quality of life (QOL) of those who suffer from these conditions. Although these symptoms may be partially ameliorated by standard treatment, including hormonal replacement therapy, many patients complain of residual symptoms. Therefore, patients’ subjectively-perceived conditions should be elicited and monitored at regular intervals in order to evaluate whether treatment is successful or not. Conditions to be addressed include patients’ physical, emotional, and social functioning, and satisfaction in daily life.

Generic or disease-specific QOL scales can be used to measure QOL in patients with hypopituitarism. Generic scales include the Nottingham Health Profile [2], the Psychological General Well-Being index [3], and the MOS Short Form 36-item Health Survey (SF-36) [4]–[6]. Some subjective patient-reported outcome measures, such as the Quality of Life - Assessment of Growth Hormone Deficiency in Adults (QoL-AGHDA) [7], [8], and the Questions on Life Satisfaction (QLS) [9], are available to evaluate impacts of hypopituitarism. We have previously evaluated both SF-36 and Qol-AGHDA in Japanese patients with growth hormone deficiency [10]. However, using these measures, we could not detect changes in QOL between patients treated with either growth hormone or a placebo. Therefore, these QOL measures may not fully evaluate the entire range of quality of life issues which patients with hypopituitarism may experience, because they emphasize symptoms induced by a particular hormone deficiency, or particular aspects of a condition, such as influence on mental state.

We therefore organized a research group to develop a new, disease-specific, psychometrically valid, patient-reported outcome measure, named the Adult Hypopituitarism Questionnaire (AHQ), in order to multilaterally and exhaustively evaluate the daily and social lives, as well as the physical and mental functioning, of patients with hypopituitarism and diabetes insipidus.

## Methods

The development of the AHQ was conducted in a standardized manner, using an accepted measure development methodology which included item development, pilot testing, and psychometric validation [11]. The study was approved by the Ethics Committee of each facility, and all participants gave written, informed consent prior to interviews or survey participation. Personally identifiable information, such as names, phone numbers, and addresses, was not collected from participants in order to fully protect their privacy.

### Item Development and Cognitive Debriefing

Questionnaire items were generated through a multi-step process: 1) review of relevant measures and related papers; 2) patient interviews; 3) examination by the research group; and 4) cognitive debriefing of a small number of patients [12].

A pool of 70 items, which consisted of candidate items that reflected the construct concept of the AHQ, was generated, primarily through patient interviews by experts and review of relevant literatures. Twelve patients with hypopituitarism were recruited and interviewed by the authors, who have experience in questionnaire development. The main purpose of these patient interviews was exploration of patient-perceived psychological and physical impacts of the disease and its treatment. This included impacts on social, psychological, and physical functioning, as well as anxiety about the future. Data obtained from these patient interviews were sorted and qualitatively analyzed to examine whether each item reflected the construct concept. The research group then selected items based on the construct concept, while taking precautions not to omit necessary concepts. A list of 81 draft items was produced. Cognitive debriefing of the preliminary AHQ was conducted with a small number of patients to assess patients’ interpretations of the questions (26 patients in the first pilot test and 13 patients in the second pilot test). These patients were asked to complete the preliminary AHQ, and were then interviewed about its comprehensiveness, relevance, and clarity of expression. Due to the limited number of patients who could participate in the pilot tests, we did not prevent patients from participating in both pilot tests.

### Psychometric Validation

A patient survey was conducted to collect answers to each question for psychometric validation. The reliability and validity of the AHQ were then psychometrically tested using the collected questionnaires.

#### 1. Participants and survey procedure

The participants in the survey were both male and female Japanese patients over 18 years old with (pan)hypopituitarism, diabetes insipidus, hypogonadotropic hypogonadism, or isolated hormone deficiency, who could understand the questionnaire and fill in their answers without assistance.

Overall, 203 participants were recruited at seven medical facilities, and were asked to complete and return the survey questionnaire to an independent research office (CLINICAL STUSY SUPPORT, Inc., Nagoya, Japan). Of these, 108 participants whose clinical conditions were stable were asked to answer the questionnaire a second time, after an interval of 1 day, to assess the test-retest reliability of the AHQ. Ten participants who had not previously received replacement therapy and who were to start therapy were asked to answer the questionnaire twice; before replacement therapy, and again 3 months or later after beginning therapy. This group is referred to as the “before-and-after-therapy” group.

In addition to the AHQ, the survey questionnaire included the SF-36v2 (Medical Outcomes Study Short Form-36 ver. 2, Japanese edition), which is a comprehensive index of health-related QOL, in order to concurrently assess the validity of the AHQ. The SF-36v2 consists of 8 domains [13]: physical functioning, role physical, bodily pain, social functioning, general health perceptions, vitality, mental health, and role emotional. The scores are expressed in two summary scores, a physical component summary score and a mental component summary score. Scores of physical functioning, role physical, and bodily pain contribute to the physical component summary; scores of role emotional, social functioning, and mental health contribute to the mental component summary; and scores of social functioning, vitality, and general health perceptions contribute to both.

#### 2. Statistical methods for psychometric testing

Demographic and clinical variables of the participants were summarized using descriptive analyses. In the item analysis, any item which met the following conditions was deleted: 1) any item whose floor effect or ceiling effect was 80% or higher; 2) one of any two items whose correlation coefficient was 0.8 or higher; 3) if the correlation coefficient between each item and the total score, excluding an item, was very low compared to that of other items.

For reliability, internal consistency and reproducibility were examined. With regard to internal consistency, the homogeneity of the question items in each domain was evaluated using Cronbach’s α coefficient. A coefficient of 0.7 or higher is preferred for a questionnaire to be internally consistent [14]. For reproducibility, the two sets of answers from the patients in the test-retest group whose clinical conditions were stable were examined using the intraclass correlation coefficient. A coefficient of 0.7 or higher was considered evidence of acceptable test-retest reliability [12].

With regard to validity, construct validity (domain structure), concurrent validity, and known-group validity were examined. For construct validity, factor analysis was performed using the principal factor method with a promax rotation to test the hypothesized domain structure. Exploratory factor analysis was also performed to examine subdomain structure, although the AHQ was developed assuming two domains. Three gender-specific items (“beards”, “erectile dysfunction”, and “menses”) were excluded from factor analysis. Concurrent validity was evaluated using Pearson’s product-moment correlation coefficient with SF-36v2. We hypothesized that the SF-36v2 sub-domains belonging to the mental component summary would correlate more strongly with the AHQ sub-domains belonging to the psycho-social domain than with those belonging to the physical domain. Likewise, we anticipated that the SF-36v2 sub-domains belonging to the physical component summary would correlate more strongly with the AHQ sub-domains belonging to the physical domain. According to the criterion of correlation strength in the psychometric validation proposed by Cohen [15], the correlation coefficient was judged as follows: 0.1 = weak correlation; 0.3 = medium correlation; and 0.5 = strong correlation. For known-group validity, relationships between selected clinical variables and the domain score were examined using a t-test. For responsiveness, a change in the mean score before and after replacement therapy in the before-and-after-therapy group was examined using a paired t-test.

AHQ item scores were transformed into a scale of 0 to 100, with higher scores indicating better patient condition. The sub-domain score was determined to be the mean score of attributive question items. When a missing response was found to a question item attributed to a sub-domain, the following procedures were employed: 1) when the number of items with a missing response in a sub-domain was less than 50% of the total number of items in the sub-domain, the mean was calculated by imputing the missing responses based on the mean of the non-missing items; 2) when the number of items with a missing response in a sub-domain was more than 50% of the total number of items in the sub-domain, the sub-domain score was not calculated. If a sub-domain score was not available, the domain score was not calculated. All statistical tests were two-tailed, and the level of significance was set at 5%.

## Results

### Item Development and Cognitive Debriefing

A total of 12 participants with panhypopituitarism and/or diabetes insipidus were interviewed in September 2004 and February 2005 to determine the question items, and seventy items were pooled as potential questions. After review by the research group, some questions were added, and 81 items covering four aspects (social functioning, mental functioning, physical functioning and condition, and anxiety about the future) were finally generated. A 7-point (0 to 6) Likert scale was employed as the response option under the assumption of an equally spaced distance between response choices.

A pilot test for cognitive debriefing was performed with 26 participants in October 2005 to examine content validity of the preliminary questionnaire in regards to factors such as relevance and clarity of language. The mean age of the patients was 47.4 years (range: 22–80), and 12 of the participants were male (46.2%). The mean time required to answer all the questions was 11.7 minutes (range: 5–35). The question items were considered to be easily understandable because when surveyed, the participants did not make any particular comments indicating difficulty answering the questionnaire as a whole, although we decided to make minor changes to some questions in response to patients’ suggestions. The second pilot test was conducted with 13 participants from November to December 2005, because the answer scale was partially modified for the questions asking about the participants’ conditions. More than 80% of the participants could easily answer the questions, indicating that modification of the answer scale did not have a negative impact on the ability of the participants to answer the questions. Finally, an 81-item provisional questionnaire was determined. Based upon theoretical considerations, a two-domain structure, with psycho-social and physical domains, was adopted.

### Psychometric Validation

From February 2006 to October 2008, answered questionnaires from 203 participants were collected, and 196 of the fully answered questionnaires were subjected to item analysis. Of these, 192 questionnaires accompanied by the background characteristics of the participants were subjected to reliability and validity testing, except for exploratory factor analysis. Exploratory factor analysis was performed on 228 questionnaires received by October 2008, in order to examine sub-domain structure.

#### 1. Backgrounds of the participants

Of the 192 questionnaires, 96 were collected from male participants (50.0%). The median age was 42.5 years (range: 18–82); mean height, 161.0 cm (standard deviation [SD]): 9.6); mean body weight, 62.1 kg (SD: 13.2); and mean body mass index (BMI), 23.8 kg/m^2^ (SD: 4.0). The deficient hormones were GH (79.7%), LH/FSH (79.2%), TSH (83.3%), ACTH (79.2%), PRL (19.8%), and ADH (26.6%). Pituitary adenoma was the most common causative disease (n = 48, 32.0%). [Table 1].

**Table 1:** Clinical characteristics of psychometric validation cohort (n = 192).

Age (yr)	median 42.5 (range: 18–82)
Gender (male: female)	96∶96
Height (cm)	161.0±9.6 (mean±SD)
Weight (kg)	62.1±13.2 (mean±SD)
Abdominal circumference (cm)	median 81.7 (range: 58.0–125.0)
Body Mass Index (kg/m^2^)	23.8±4.0 (mean±SD)
Hormone Deficiency (n, %)	
GH	153 (79.7%)
LH/FSH	152 (79.2%)
TSH	160 (83.3%)
ACTH	152 (79.2%)
PRL	38 (19.8%)
ADH	51 (26.6%)
Diagnosis of Underlying Causes (n, %)	
Idiopathic and unknown	42 (21.9%)
Organic	150 (78.1%)
Pituitary adenoma †	48
Craniopharyngioma	26
Germinoma	20
Sheehan’s syndrome	13
Birth Injury	10
Others ‡	33

† Including clinically non-functioning (n = 25); GH-secreting (n = 13); PRL-secreting (n = 7); ACTH-secreting (n = 2); TSH-secreting (n = 1).

‡ Including pituitary stalk transection (n = 8); Hypothalamic tumor (n = 7); Rathke’s cleft cyst (n = 6); Pituitary tumor (n = 6); Hypophysitis (n = 4), etc.

**Table 2:** Exploratory factor analysis (n = 228).

Psycho-social domain	Factor loading							
	1	2	3	4	5		6	
Factor1: Depressed mood								
Q20 Do you feel down easily?	0.77	−0.01	0.09	0.01	−0.11		0.23	
Q22 Does your mood change easily?	0.36	0.06	0.16	−0.01	0.02		0.34	
Q27 Are you easily irritated?	0.63	−0.03	0.00	0.08	0.14		0.04	
Q28 Do you become sad easily?	0.88	−0.09	−0.06	0.02	0.02		0.12	
Q29 Do you feel depressed?	0.78	0.03	−0.01	0.01	0.04		0.11	
Q12 Do you get tired easily after events or activities?	0.43	0.42	0.16	−0.10	0.07		−0.11	
Q13 Do you easily get tired for no reason?	0.49	0.47	0.12	−0.01	−0.01		−0.19	
Q30 Do you feel uneasy when you think about your future?	0.48	0.06	−0.10	0.00	0.36		0.08	
Factor 2: Limitation in social activities								
Q1 Are you reluctant to spend the night at any place other than your home?	0.04	0.50	0.01	0.06	0.08		0.19	
Q2 Are you reluctant to go out?	0.00	0.79	0.01	0.01	−0.11		0.19	
Q3 Do you have trouble finding a toilet when you go out?	−0.14	0.32	0.17	0.06	0.06		0.18	
Q4 Do you sometimes cancel your plans on the day of the event?	−0.08	0.56	−0.11	0.08	0.03		0.29	
Q9 Do you feel that the things you want to do are limited?	0.03	0.44	−0.04	0.00	0.23		0.31	
Q10 Are your work and activities hindered by treatment?	−0.09	0.58	0.01	−0.08	0.35		−0.02	
Q14 Are you reluctant to go out on holidays?	0.09	0.62	0.09	0.06	−0.02		0.03	
Q11 Do you have trouble doing housework or working?	0.33	0.52	0.17	−0.03	−0.01		−0.14	
Factor 3: Vigor								
Q15 Do you find it difficult to get things done?	0.00	0.14	0.75	0.07	−0.02		0.05	
Q16 Do you find it difficult to maintain concentration?	−0.02	−0.10	0.94	0.00	0.11		−0.02	
Q17 Do you give up easily?	0.08	0.12	0.76	0.03	0.01		−0.04	
Q18 Do you get tired of things easily?	−0.03	−0.05	0.59	−0.03	0.13		0.28	
Q19 Are you uninterested in most things?	0.05	0.22	0.38	−0.05	−0.12		0.40	
Q21 Do you have trouble finding motivation?	0.48	0.10	0.46	−0.05	−0.07		0.06	
Factor 4: Sleep								
Q23 Do you have difficulty going to sleep?	−0.01	0.00	0.05	0.72	−0.03		0.02	
Q24 Do you get sleepy during the day?	0.31	0.07	0.23	0.13	0.10		−0.14	
Q25 Are you unable to sleep well?	0.05	0.01	0.00	0.74	0.07		−0.09	
Q26 Do you have difficulty waking up?	0.21	0.15	0.01	0.43	−0.05		0.05	
Factor 5: Anxiety about treatment								
Q31 Are you concerned about financial issues?	0.25	0.18	−0.14	0.02	0.50		0.03	
Q32 Do you have concerns about continuing treatment?	0.01	0.08	0.10	0.03	0.74		−0.03	
Q33 Do you feel that others do not understand your symptoms?	0.10	0.13	0.05	−0.04	0.43		0.05	
Q34 Do you find it troublesome to continue treatment?	−0.02	−0.15	0.20	0.02	0.61		0.13	
Factor 6: Interpersonal relationships								
Q5 Do you find it difficult to socialize with others?	−0.05	0.22	0.06	0.04	0.02		0.67	
Q6 Do you get worried about your appearance?	0.30	−0.04	−0.02	−0.01	0.10		0.41	
Q7 Do you feel that you have few friends?	0.08	0.05	0.00	−0.10	0.04		0.71	
Q8 Is your mood easily influenced by others?	0.43	−0.06	−0.02	0.06	0.03		0.47	
Factor 1: Control of body temperature								
Q37 Do you have poor circulation?	0.85	−0.12	−0.01	0.02	0.03		−0.16	0.04
Q38 Does your body temperature tend to vary?	0.77	−0.01	0.19	−0.05	−0.02		−0.07	0.06
Q39 Are you sensitive to high temperatures?	0.36	0.07	0.09	0.10	−0.03		0.14	0.05
Q40 Are you sensitive to low temperatures?	0.70	0.11	0.01	0.03	0.10		−0.04	−0.08
Q70 Do you tend to feel chilly or dizzy?	0.58	0.19	0.10	0.07	−0.03		0.00	−0.06
Q71 Do you often forget things?	0.26	0.23	−0.08	0.04	0.18		0.02	0.05
Factor 2: Physical strength								
Q35 Do you get tired easily?	0.22	0.30	0.47	0.01	−0.04		0.15	−0.07
Q36 Do you lack stamina?	0.27	0.40	0.30	−0.05	−0.03		0.18	−0.01
Q58 Do you feel unwell?	−0.05	0.44	0.51	−0.01	0.08		−0.04	−0.04
Q63 Are you unable to walk long distances?	0.02	0.82	−0.10	0.02	0.07		0.01	0.05
Q64 Do you have difficulty going up stairs?	0.02	0.82	−0.04	0.02	0.04		0.01	0.00
Factor3: Immunity, digestive tract, and musculoskeletal system								
Q52 Do you tend to catch colds easily?	−0.01	−0.13	0.49	0.24	0.02		0.10	−0.02
Q55 Do you tend to get canker sores?	−0.03	−0.34	0.55	0.28	0.05		0.02	0.02
Q56 Do you have a weak appetite?	−0.11	0.10	0.54	0.07	0.18		−0.40	0.11
Q57 Do you feel nauseous?	−0.02	0.23	0.49	−0.01	0.18		−0.17	−0.09
Q50 Do you have stiff shoulders?	0.16	0.06	0.47	−0.11	−0.10		0.11	0.10
Q53 Do your joints ache?	0.05	0.20	0.39	0.12	0.00		−0.02	0.00
Q74 Do you get headaches?	0.15	−0.04	0.51	0.07	−0.06		0.03	0.07
Factor 4: Urination								
Q65 Do you urinate frequently during the day?	0.07	−0.03	0.02	0.77	0.07		0.06	0.01
Q66 Do you wake up many times in the night to urinate?	−0.05	0.14	0.09	0.72	0.00		−0.08	0.08
Q67 Do you discharge a large amount of urine throughout the day?	0.04	−0.01	0.07	0.88	−0.01		−0.04	0.02
Q68 Do you tend to get thirsty?	0.01	0.08	0.14	0.56	0.00		0.20	−0.05
Factor 5: Skin condition and visual acuity								
Q60 Do your wounds tend to take a long time to heal?	−0.06	0.23	−0.05	0.08	0.59		−0.02	0.03
Q61 Do you tend to get bruises?	−0.05	0.08	−0.04	0.12	0.56		0.07	0.01
Q62 Do you have decreased skin elasticity?	0.06	0.31	−0.10	−0.04	0.50		−0.01	0.11
Q59 Are you unable to talk loudly?	−0.01	0.25	0.17	0.04	0.35		−0.08	−0.15
Q72 Do you have a narrow field of vision?	0.01	−0.08	0.12	0.01	0.49		0.05	0.08
Q73 Has your eyesight become worse?	0.09	−0.13	0.23	−0.08	0.48		0.09	0.04
Q54 Do your eyes get tired easily?	0.14	−0.11	0.42	−0.11	0.42		0.12	−0.07
Q41 Do you tend not to sweat?	0.10	0.12	0.06	0.13	0.25		0.00	0.00
Factor 6: Body weight								
Q44 Do you have a big waist?	−0.06	−0.04	0.03	−0.03	0.07		0.82	0.07
Q45 Do you gain weight easily?	−0.07	−0.01	0.06	−0.02	0.02		0.92	−0.03
Q46 Are you too heavy?	−0.16	0.03	0.06	0.03	0.01		0.88	−0.01
Q51 Do you have swelling in your face and legs?	0.09	0.14	0.16	−0.11	0.16		0.32	0.12
Q69 Do you have an excessive appetite?	0.19	0.04	−0.26	0.21	0.01		0.58	−0.02
Factor 7: Sexual function								
Q42 Do you lack sexual desire?	−0.04	0.17	0.26	0.00	−0.10		0.03	0.62
Q43 Do you lack interest in the opposite sex?	−0.10	0.29	0.15	0.01	−0.12		0.01	0.66
Q47 Do you have little hair under your armpits and in your pubic area?	0.12	−0.14	−0.09	0.06	0.13		0.05	0.71
Q48 Do you have little body hair?	0.01	−0.08	−0.09	0.04	0.15		−0.06	0.80
Q49 Do you have little hair on your head?	0.16	−0.01	−0.02	−0.10	0.24		0.08	0.24

Note: The following 3 gender specific items are not included in the factor analysis: Q75 (Do you have little facial hair?), Q76 (Are you impotent?), (Are you impotent?), and Q77 (Do you no longer have menstrual periods?).

#### 2. Item analysis

As a result of the item analysis of 196 answered questionnaires, floor and ceiling responses were not observed in the distribution of answers. With regard to correlations between items, three item pairs that exhibited correlation coefficients of 0.8 or higher were found. Since these items apparently dealt with similar concepts, the item which was considered to be most understandable was retained. There was only one item (“frequent perspiration”) for which the correlation coefficient between the score of this item and the total score of items except this item was notably lower than the correlation coefficient between the score of the other individual items and the total score of items except that individual item (its correlation coefficient was 0.16). The content of the question regarding “frequent perspiration”, from which this item was derived, was not considered to be especially important to patients with hypopituitarism, and hence it was deleted. On the basis of the results obtained from item analysis, 4 items were deleted and 77 items were retained in the questionnaire.

**Table 3:** Mean score, Cronbach’s alpha, and intraclass correlation coefficient.

AHQ Domains and Sub-domains		Mean Score(n = 192)	Cronbach’s alpha coefficient (n = 192)	Intraclass correlation coefficient (n = 108)
	Psycho-social	56.6	0.96	0.91
	Physical	61.4	0.95	0.95
Psycho-social	Factor1: Depressed mood	52.7	0.93	0.90
	Factor 2: Limitation in social activities	63.6	0.90	0.90
	Factor 3: Vigor	57.2	0.92	0.90
	Factor 4: Sleep	55.7	0.72	0.78
	Factor 5: Anxiety about treatment	55.7	0.72	0.77
	Factor 6: Interpersonal relationships	51.4	0.84	0.88
Physical	Factor 1: Control of body temperature	53.7	0.89	0.92
	Factor 2: Physical strength	55.6	0.89	0.94
	Factor 3: Immunity, digestive tract, and musculoskeletal system	68.0	0.73	0.92
	Factor 4: Urination	61.5	0.87	0.94
	Factor 5: Skin condition and visual acuity	65.3	0.78	0.90
	Factor 6: Body weight	49.7	0.87	0.91
	Factor 7: Sexual function	69.8	0.73	0.86

Note: Higher scores indicate better condition.

#### 3. Factor analysis

The AHQ was developed assuming two domains; psycho-social and physical. Factor analysis was performed using the principal factor method and a promax rotation to examine the domain structure, and almost all the items were classified into the assumed domains. Of the items that had been assumed to be classified into the physical domain, 4 items (items 36, 44, 45, and 58) showed approximately the same factor loading values in both the domains, and 1 item (item 59) was strongly regressed to the psycho-social domain. However, based on their conceptual interpretability, all the items were incorporated into the physical domain.

**Table 4:** Pearson’s correlation coefficients between AHQ and SF36v2 scores.

		SF36v2 Summary Score		SF36v2 Sub-domains							
		PhysicalComponentSummary	MentalComponentSummary	PhysicalFunction(PF)	RolePhysical(RP)	BodilyPain (BP)	SocialFunctioning(SF)	General HealthPerception (GH)	Vitality(VT)	RoleEmotional(RE)	MentalHealth(MH)
AHQ domains	Psycho-social	0.35	0.69	0.34	0.47	0.28	0.55	0.61	0.68	0.54	0.65
	Physical	0.46	0.49	0.48	0.48	0.39	0.44	0.52	0.54	0.51	0.48
Psycho-social	Factor 1: Depressed mood	0.31	0.72	0.30	0.44	0.29	0.53	0.59	0.68	0.50	0.67
	Factor 2: Limitation in social activities	0.44	0.60	0.40	0.52	0.31	0.58	0.55	0.62	0.55	0.58
	Factor 3: Vigor	0.32	0.52	0.32	0.41	0.13	0.43	0.47	0.56	0.49	0.51
	Factor 4: Sleep	0.20	0.44	0.20	0.30	0.21	0.25	0.45	0.41	0.36	0.40
	Factor 5: Anxiety about treatment	0.19	0.55	0.20	0.30	0.23	0.37	0.52	0.50	0.35	0.45
	Factor 6: Interpersonal relationships	0.14	0.54	0.16	0.27	0.13	0.40	0.38	0.47	0.33	0.50
Physical	Factor 1: Control of body temperature	0.29	0.39	0.31	0.30	0.31	0.30	0.32	0.40	0.38	0.40
	Factor 2: Physical strength	0.58	0.54	0.63	0.57	0.45	0.52	0.63	0.66	0.56	0.52
	Factor 3: Immunity, digestive tract, and musculoskeletal system	0.33	0.40	0.36	0.34	0.38	0.32	0.42	0.42	0.38	0.37
	Factor 4: Urination	0.36	0.25	0.37	0.37	0.23	0.27	0.37	0.27	0.37	0.31
	Factor 5: Skin condition and visual acuity	0.42	0.41	0.36	0.47	0.31	0.43	0.42	0.43	0.51	0.44
	Factor 6: Body weight	0.16	0.23	0.18	0.19	0.22	0.16	0.20	0.29	0.19	0.17
	Factor 7: Sexual function	0.25	0.27	0.31	0.30	0.15	0.26	0.34	0.30	0.27	0.26

Note: All correlation coefficients are p<0.05.

Exploratory factor analysis was performed on 228 questionnaires that had been accumulated by October 2008. The psycho-social domain was divided into six sub-domains. These six sub-domains were labeled “depressed mood”, “limitation in social activities”, “vigor”, “sleep”, “anxiety about treatment”, and “interpersonal relationships”, on the basis of interpretability of the attributive items. The physical domain was divided into seven sub-domains. They were termed “control of body temperature”, “physical strength”, “immunity, digestive tract, and musculoskeletal system”, “urination”, “skin condition and visual acuity”, “body weight,” and “sexual function.” [Table 2].

#### 4. Reliability

Cronbach’s α coefficient ranged from 0.72 to 0.93 for the psycho-social sub-domains, and from 0.73 to 0.89 for the physical sub-domains, indicating acceptable internal consistency. Regarding reproducibility, the intraclass correlation coefficient ranged from 0.77 to 0.90 for the psycho-social sub-domains, and from 0.86 to 0.94 for the physical sub-domains. Their reproducibility was considered sufficient. [Table 3].

**Figure 1:**
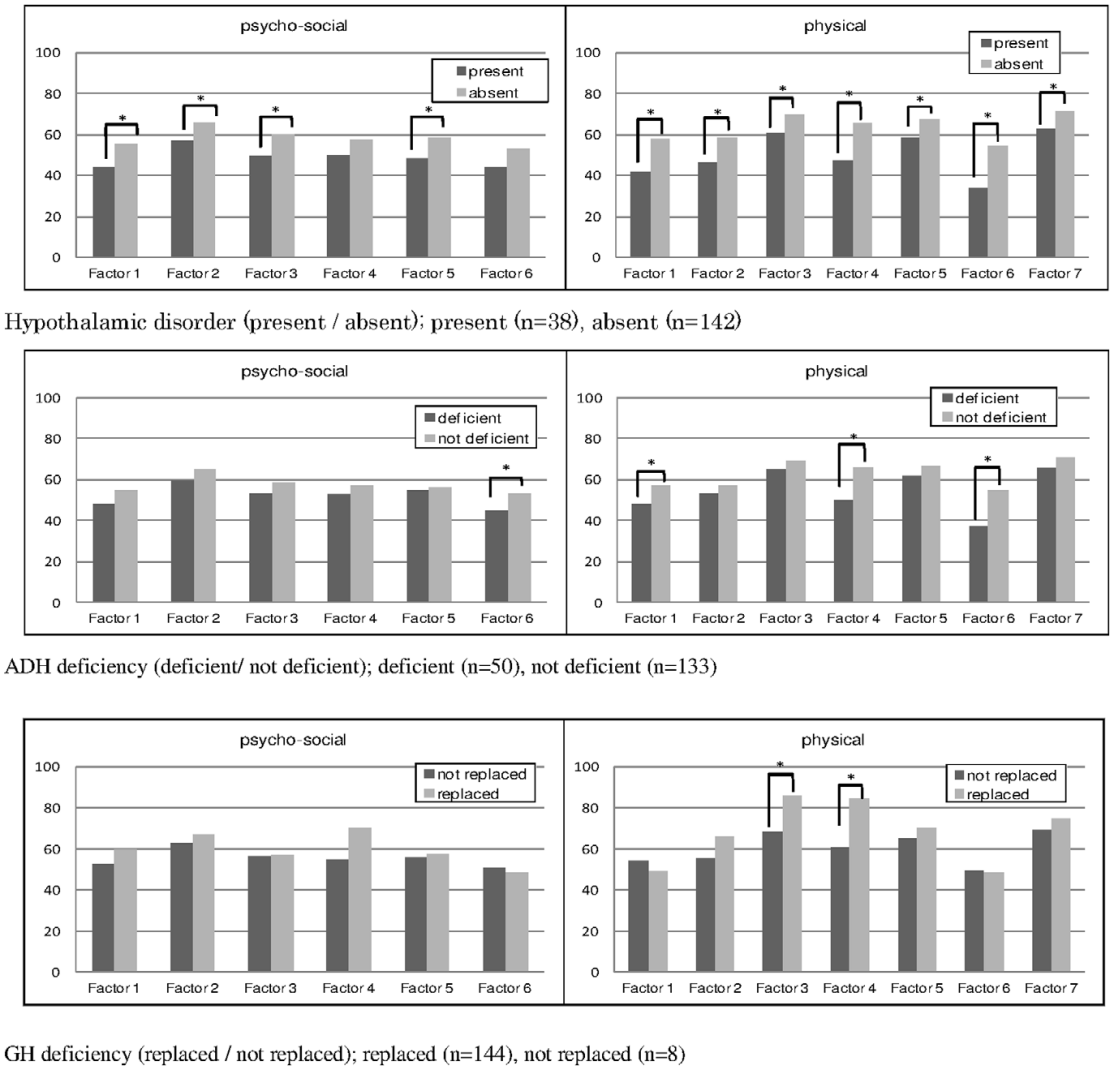
Sub-domain scores by clinical variables that might affect the scores. Note 1: All p values were calculated by t-test, and asterisks indicate statistically significant differences (P<0.05) between groups. Note 2: Sub-domain names are as follows: Psycho-social domain (Factor 1: Depressed mood, Factor 2: Limitation in social activities, Factor 3: Vigor, Factor 4: Sleep, Factor 5: Anxiety about treatment, Factor 6: Interpersonal relationships) Physical domain (Factor 1: Control of body temperature, Factor 2: Physical strength, Factor 3: Immunity, digestive tract, and musculoskeletal system, Factor 4: Urination, Factor 5: Skin condition and visual acuity, Factor 6: Body weight, Factor 7: Sexual function).

#### 5. Concurrent validity

The correlation coefficients between the AHQ sub-domains and the eight domains in the SF-36v2 were calculated in order to examine concurrent validity. The AHQ sub-domains correlated moderately with all of the SF-36v2 sub-domains, ranging from 0.13 to 0.68. As hypothesized, the physical component summary of the SF-36v2 correlated more strongly with the physical domain of the AHQ, and the mental component summary correlated more strongly with the psycho-social domain of the AHQ, although correlation coefficients were not compared statistically. [Table 4].

#### 6. Known-group validity

The relationship between clinical variables that may affect scores was examined. AHQ sub-domain scores clearly differentiated between the presence and absence of hypothalamic disorder with significant differences noted in 11 sub-domains. The patients with ADH deficiency showed significantly lower scores in “interpersonal relationships”, “control of body temperature”, “urination”, and “body weight”. For GH deficiency, statistical significance was shown only in “immunity, digestive tract, and musculoskeletal system” and “urination”. [Figure 1].

#### 7. Responsiveness

Changes in the mean score before and after hormone replacement therapy were examined using ten sets of questionnaires for which background information of the participants could be collected. The scores in all the sub-domains were higher after therapy, but a statistically significant difference was observed only in “depressed mood”, “vigor”, and “physical strength” (p’s = 0.03, 0.01, and 0.04, respectively; paired t-test).

## Discussion

We developed and validated the Adult Hypopituitarism Questionnaire (AHQ) as a new, disease-specific, self-administered measure for evaluating QOL in adult patients with hypopituitarism.

On the basis of psychometric testing, the AHQ was judged to be reliable and valid as a questionnaire for patients with hypopituitarism. Regarding reliability, good to excellent internal consistency and reproducibility were observed in all the sub-domains. Regarding validity, concurrent validity was suggested since the SF-36v2 sub-domains correlated more strongly with the related AHQ domains, as hypothesized, although a statistical comparison was not conducted. For known-group validity, relevance was exhibited between the sub-domain score and the clinical variables that might affect the scores, such as the presence or absence of hypothalamic disorder or ADH deficiency, indicating that the disordered or deficient group showed lower scores. With regard to GH deficiency, significant differences were observed in only two physical sub-domains. However, it is important to note that these results about known-group validity cannot be interpreted with confidence, because they were not obtained through a randomized placebo-controlled trial. It also remains controversial whether amelioration of GH deficiency-induced impairments translates into clinically meaningful improvement in physical function and QOL [16]. Further clinical studies using randomized, placebo-controlled designs and involving substantial numbers of patients would be required to demonstrate the benefits of this treatment on improving QOL.

Regarding factor analysis, the items “lack of physical strength”, “big waist size”, “easy to get fat”, “poor physical condition”, and “cannot speak loudly” were not clearly classified into the assumed physical domain. Despite the unexpected loading of these items, the AHQ still showed sufficient internal consistency; therefore, they could be incorporated into the physical domain based on their conceptual interpretability.

Although the 77 questions included in the AHQ seem like a large number, and might result in long completion time, the AHQ is able to detect various impacts of the disease and its treatments, in multiple aspects. Since most of the currently available measures emphasize particular aspects such as mental status, the AHQ can assess impacts that are not detected by existing measures. It is also important when treating chronic diseases such as hypopituitarism that the patient’s condition, including QOL, be monitored longitudinally. The AHQ might help clinicians understand the severity of the disease and detect changes caused by treatment, as well as allow them to compare efficacy between treatments.

The AHQ was developed in Japanese, for Japanese patients with hypopituitarism. However, since the AHQ does not contain items that are specifically related to Japanese culture, it could be translated and used internationally. We note that the English version of the AHQ shown in this paper has not been linguistically validated. To develop a translated edition, the content must be translated in a linguistically appropriate manner. Moreover, the psychometric properties of the translated edition need to be assessed. Ideally, the translated edition should have the same domain structure as that of the original edition [17]–[19], enabling researchers to compare internationally obtained data.

Several limitations of this study should be noted. First, the survey was conducted at only eight hospitals, raising the issue of generalizability of the findings. To minimize this concern, broad eligibility criteria (over 18 years of age, with panhypopituitarism, hypogonadotropic hypogonadism, or isolated hormone deficiency) were employed. However, we note that there are a relatively small number of pituitary adenoma patients and a high number of germinoma and Sheehan’s syndrome patients in this study. Second, it should also be noted that further assessment is necessary with respect to responsiveness to treatment. In this study, only 10 participants were examined for responsiveness, and there were no randomly assigned control groups. A larger number of participants need to be assessed in order to draw a firm conclusion, although a questionnaire with good reliability and validity is most likely to detect any clinically meaningful changes, in general [20]. Another concern is the one-day interval of the test-retest survey. When a short test-retest interval is employed, it is possible that patients may remember their responses and respond based on recall. In this study, however, the large number of questions in the AHQ and the use of a 7-point Likert scale may have prevented participants from responding based on recall to some extent.

This study did not investigate the relative performance of the AHQ compared with other available measures; therefore, it cannot be concluded which measure is most appropriate in a given research or clinical setting.

Based on the findings of this study, the AHQ is a potentially useful tool for estimating hypopituitarism-related symptoms, for monitoring QOL as a part of clinical management, and for the evaluation of treatment outcomes.
